#  Aging and the number sense: preserved basic non-symbolic numerical processing and enhanced basic symbolic processing

**DOI:** 10.3389/fpsyg.2015.00999

**Published:** 2015-07-15

**Authors:** Jade E. Norris, William J. McGeown, Chiara Guerrini, Julie Castronovo

**Affiliations:** ^1^Department of Psychology, University of HullHull, UK; ^2^School of Psychological Sciences and Health, University of StrathclydeGlasgow, UK

**Keywords:** aging, numerical cognition, number sense, approximate number system, exact number system, non-symbolic numerical processing, symbolic numerical processing, quantity discrimination

## Abstract

Aging often leads to general cognitive decline in domains such as memory and attention. The effect of aging on numerical cognition, particularly on foundational numerical skills known as the number sense, is not well-known. Early research focused on the effect of aging on arithmetic. Recent studies have begun to investigate the impact of healthy aging on basic numerical skills, but focused on non-symbolic quantity discrimination alone. Moreover, contradictory findings have emerged. The current study aimed to further investigate the impact of aging on basic non-symbolic and symbolic numerical skills. A group of 25 younger (18–25) and 25 older adults (60–77) participated in non-symbolic and symbolic numerical comparison tasks. Mathematical and spelling abilities were also measured. Results showed that aging had no effect on foundational non-symbolic numerical skills, as both groups performed similarly [RTs, accuracy and Weber fractions (*w*)]. All participants showed decreased non-symbolic acuity (accuracy and *w*) in trials requiring inhibition. However, aging appears to be associated with a greater decline in discrimination speed in such trials. Furthermore, aging seems to have a positive impact on mathematical ability and basic symbolic numerical processing, as older participants attained significantly higher mathematical achievement scores, and performed significantly better on the symbolic comparison task than younger participants. The findings suggest that aging and its lifetime exposure to numbers may lead to better mathematical achievement and stronger basic symbolic numerical skills. Our results further support the observation that basic non-symbolic numerical skills are resilient to aging, but that aging may exacerbate poorer performance on trials requiring inhibitory processes. These findings lend further support to the notion that preserved basic numerical skills in aging may reflect the preservation of an innate, primitive, and embedded number sense.

## Introduction

The current study aims to investigate the effect of healthy aging on basic numerical processes, often referred to as the number sense (Dehaene, 2009), by assessing the foundational non-symbolic and symbolic numerical skills of healthy older adults in comparison to younger adults with the use of classic non-symbolic and symbolic numerical comparison tasks. It is unclear whether basic numerical abilities remain intact in aging, similar to semantic knowledge, vocabulary, and reasoning (Hedden and Gabrieli, 2004; Salthouse, 2009), or decline as do working and episodic memory, attention, and executive processes (El Yagoubi et al., 2005; Lemaire and Lecacheur, 2007; Deary et al., 2009; Salthouse, 2009). Research into the cognitive implications of aging is vital in understanding differences between normal and pathological aging, and in determining whether interventions may slow decline (Dixon et al., 2004; Salthouse, 2009). Numerical cognition in healthy aging has been under-researched, particularly in terms of basic foundational numerical skills. These abilities belong to the number sense, an umbrella term for both symbolic and non-symbolic foundational number skills.

There are two forms of basic numerical processing: non-symbolic (quantities, such as sets of dots) and symbolic (numerical symbols, such as Arabic digits). Both non-symbolic and symbolic numerical processing are associated with the ‘Number Sense’ (Dehaene, 1997, 2009; Feigenson et al., 2004; Verguts and Fias, 2004), and both play an important role in the acquisition of more advanced numerical and arithmetical skills, such as mathematical achievement (e.g., De Smedt et al., 2009; Holloway and Ansari, 2009; Piazza et al., 2010). Foundational non-symbolic numerical skills are usually referred to as the approximate number system (ANS: Halberda et al., 2008). The ANS is defined as the ability in infants, children, adults, and some animal species to approximate non-symbolic numerical magnitudes without counting, for example when estimating the amount of sweets in a jar, or choosing the shortest queue at the supermarket (Gallistel and Gelman, 2000; Feigenson et al., 2004; Piazza et al., 2004; Dehaene, 2009; Desoete et al., 2009; Izard et al., 2009; Piazza and Izard, 2009). This primitive non-symbolic numerical system is thought to be innate, originating from evolutionary systems (Dehaene, 1997; Feigenson et al., 2004; Piazza and Izard, 2009; Park et al., 2012). The ANS has been found to obey Weber’s law, indicated by the consistent observation of size and distance effects in quantity discrimination tasks (respectively, slower and less accurate responses as numerosity magnitude increases, as well as slower and less accurate responses the smaller the distance between to-be-discriminated quantities, e.g., Dehaene, 1997; Piazza and Izard, 2009; Roitman et al., 2012). ANS acuity has been measured and expressed with the Weber fraction (*w*: Halberda et al., 2008). The *w* indicates an individual’s numerical representation precision, with a higher *w* indicating a ‘noisier’ and less accurate representation (Piazza et al., 2004; Halberda et al., 2008). Several studies have demonstrated that ANS acuity improves in typical development, reflected by a decrease in *w*, with an average *w* in educated numerate adults of ∼0.15 (Lipton and Spelke, 2004; Halberda and Feigenson, 2008; Halberda et al., 2008; Dehaene, 2009; Piazza et al., 2013). Following the acquisition of symbolic numerical knowledge during development, the ANS is suggested to be progressively refined into a symbolic exact number system (ENS: Verguts and Fias, 2004; Dehaene, 2009; Castronovo and Göbel, 2012). The ENS has been defined as a later-acquired, formal, symbolic, and linear numerical system, which accounts for automatic access between symbolic numbers and their corresponding magnitude (Verguts and Fias, 2004; Dehaene, 2009). The idea of the refinement of the ANS into the ENS with development is supported by behavioral data, such as the observation of improving precise linear pattern of performance on number line tasks in children with increasing age (e.g., Opfer and Siegler, 2007; Ashcraft and Moore, 2012), and the gradual development of an automatic activation of the ENS with age during symbolic number Stroop paradigms (e.g., Rubinsten et al., 2002). Neuroimaging data further support the existence of the ENS alongside the ANS (e.g., Cohen Kadosh et al., 2011). Whilst the innate ANS has been associated with the right intra-parietal sulcus and approximate processing of non-symbolic quantities (Siegler and Opfer, 2003; Dehaene, 2009; Mazzocco et al., 2011), the acquired ENS has been associated with the left parietal lobe and symbolic number processing (Ansari et al., 2006; Cantlon et al., 2006; Piazza et al., 2007; Izard et al., 2008). The ENS also obeys Weber’s law, but is suggested to be more precise, producing less pronounced distance and size effects than non-symbolic stimuli (e.g., Buckley and Gillman, 1974).

Both non-symbolic and symbolic numerical systems have primarily been studied in children and young adults. Therefore, the impact of healthy aging on these two foundational numerical systems is not well-known. Further investigation of this issue is of clear importance, notably considering evidence that healthy aging is associated with structural changes such as gray matter atrophy and declines in regional cerebral metabolic rate for oxygen in the parietal lobes (for a review see Dennis and Cabeza, 2008), which play a crucial role in foundational numerical skills (Piazza and Izard, 2009). So far, it is unclear whether these neurological changes manifest behaviorally in terms of older adults’ basic numerical abilities. Studies in the field of numerical cognition and aging have mostly investigated high-level mathematical skills, such as counting, arithmetic problem solving, and strategies for quantification (e.g., Duverne and Lemaire, 2004; El Yagoubi et al., 2005; Gandini et al., 2008). More recently, a handful of studies have investigated more foundational skills, such as non-symbolic numerosity discrimination (Li et al., 2010; Dormal et al., 2012; Halberda et al., 2012; Cappelletti et al., 2014). However, results are contradictory, and therefore no clear conclusion can yet be drawn on the effect of aging on these basic numerical skills (see below). Moreover, to our knowledge, the question of the effect of aging on foundational symbolic numerical skills has also yet to be directly addressed.

Research on the effect of aging on symbolic numerical skills has mainly focused on exact, complex abilities such as arithmetical problem solving (e.g., Duverne and Lemaire, 2004; Lemaire and Arnaud, 2008). In an early study on aging and symbolic numerical processing, Salthouse and Kersten (1993) trained older and younger adults to recognize abstract symbols as the digits 1–9. The older group made fewer errors than the younger group in an arithmetic task using Arabic digits, but recognized the new symbols as digits less accurately than the younger adults. These results were attributed to a speed of processing decline in aging rather than to a decline in arithmetical skills (Salthouse and Kersten, 1993). However, when investigating the effect of aging on simple (single digit) and complex (three-digit numbers) arithmetical problems, Duverne and Lemaire (2004) suggest that arithmetic accuracy and the ability to choose the correct strategy declines with age, especially with more complex problems. It has since been suggested that older adults may have a smaller repertoire of strategies to solve arithmetic problems and poorer efficiency in selecting an effective strategy (Duverne and Lemaire, 2004; Lemaire and Arnaud, 2008). This is supported by ERP data, which demonstrates a left hemisphere advantage in younger adults during an arithmetic problem-verification task which is reduced in the older group (El Yagoubi et al., 2005). The authors concluded that the number of potential strategies may be reduced in the older group, whereas younger adults are able to flexibly choose the most effective strategy for the problem. Alongside the study of high-level arithmetic skills in aging, basic symbolic numerical skills were only briefly investigated in Cappelletti et al.’s (2014) study. In this recent study, older and younger participants took part in a symbolic comparison task on numbers ranging from 1 to 9 as part of a battery of arithmetical tasks. Although the results of this task were neither explicitly presented nor discussed by the authors, they seem to show no difference between older and younger adults in terms of comparison accuracy, but reveal a general slowing in the older group. No clear conclusion can be drawn from these early findings on basic symbolic numerical skills in aging, notably because of the use of a small numerical range which, considering the task’s low level of difficulty, may not allow clear dissociation between age groups. Furthermore, previous research on symbolic numerical skills in aging has also provided mixed results, with some suggesting a decline in such skills (e.g., Duverne and Lemaire, 2004; Lemaire and Arnaud, 2008), and others finding no impact of aging or superior symbolic skills in older adults (Salthouse and Kersten, 1993; Cappelletti et al., 2014). Moreover, the majority of studies have focused on higher level symbolic numerical skills, such as arithmetic problem solving, neglecting foundational symbolic abilities. Therefore, the question of the impact of aging on foundational symbolic numerical skills is still very much open and requires investigation using a classic symbolic numerical task, such as a symbolic comparison task (Moyer and Landauer, 1967), with a large enough numerical range to allow discrimination between participants’ ENS acuity.

A limited number of studies have investigated basic non-symbolic numerical processing in aging. Further, methods and stimuli used have varied widely, with some contradictory results. Firstly, some research has suggested a deterioration of basic non-symbolic numerical processing with age. Trick et al. (1996) studied the effect of aging on basic non-symbolic numerical skills with series of speeded quantity discriminations: 1 vs. 2; 3 vs. 4; 6 vs. 7; and 8 vs. 9. Participants indicated as quickly as possible whether two arrays presented for up to 7,800 ms contained an equal number or *n*+1 dots. In the subitizing range, the results suggest no decline in quantity discrimination skills with age. However, older participants performed more slowly than younger adults beyond the subitizing range. The authors concluded that these results suggest a decline in quantity discrimination speed beyond subitizing in older adults. However, further consideration of the results on accuracy presented in Figure 4 (Trick et al., 1996, p. 928) indicates higher accuracy in older compared to younger participants in the larger quantity discrimination range, possibly reflecting a speed-accuracy trade-off. On the contrary, Watson et al. (2005) found similar enumeration performance between 1 and 9 ‘Os’ in older and younger adults in both the subitizing and non-subitizing ranges. However, when distracters were added, enumeration speed decreased for the older group only, possibly due to increased visual fixations for older adults in trials containing distracters compared to those without distracters. Whilst studying estimation skills on small (20–39) and large (40–65) non-symbolic numerosities in healthy and pathological aging [Alzheimer’s disease (AD)], Gandini et al. (2009) showed that healthy aging is associated with poorer estimation speed but not accuracy, concluding that slower estimation in aging could reflect a decline in processing speed rather than a decline in numerical abilities *per se*. However, lower accuracy for older compared to younger adults on estimation of numerosities between 4 and 79 further supports a negative impact of aging on non-symbolic quantity processing (Gandini et al., 2009). In view of these initial, somewhat contradictory data, no clear conclusion can be drawn on a decline in basic non-symbolic numerical processing in aging. Further, long stimulus displays used may have encouraged enumeration, rather than measuring approximate non-symbolic numerical processing.

More recently, some authors have begun to study non-symbolic numerosity processing in aging using brief stimulus presentation times. In Li et al.’s (2010) study, older and younger participants were submitted to an estimation task on a small range (1–9), in which target numerosities were presented for 200 ms. The authors found that aging appears to be associated with poorer performance in small-quantity estimation, with older adults presenting slower reaction times (RTs) and greater response variability than the younger adults. However, since both groups presented similar accuracy overall, the authors suggested that older participants’ slower performance and higher response variability in small numerosity estimation may be better accounted for by declining peripheral cognitive processes, such as spatial selective attention and visual memory, rather than by a decline in numerical abilities specifically. In a large-scale study including more than 10,000 participants ranging from 11 to 85 years of age, Halberda et al. (2012) studied ANS acuity over the life-span with the use of ‘Panamath,’ a paradigm in which participants decide which of a set of blue and a set of yellow dots is more numerous (http://panamath.org/). Their results suggested a decrease in ANS acuity (i.e., increased *w*) from around age 30. However, the authors did not further discuss this observed age-related decline in ANS acuity.

Whilst some research suggests a decline in foundational non-symbolic numerical skills, other studies indicate that such skills are resilient to aging. Lemaire and Lecacheur (2007) investigated the effect of aging on approximation, with eye-movements recorded as younger and older adults estimated the numerosity of large sets of dots (40–460). Stimuli remained on-screen until response for up to 6 s. Results showed similar accuracy but different eye-movement patterns between groups. Older participants made more numerous but shorter fixations and scanned stimuli more broadly than younger participants. The authors suggested that older adults may use compensatory strategies during estimation to negate the impact of deteriorated vision. Such eye-movement patterns suggest that participants were likely using counting strategies rather than approximation. Dormal et al. (2012) investigated numerosity and duration processing in healthy aging and Parkinson’s disease (PD). They used a numerosity comparison task in which participants compared two series of flashing dots, ranging in numerosity from 5 to 9, and a duration comparison task in which participants compared two successive sets of flashing dots varying in duration. Although older adults made more duration comparison errors, there was no effect of aging on numerosity comparison, with older, younger and PD participants performing similarly. Further, Lambrechts et al. (2013) studied spatial and duration processing in older and younger adults using comparison and bisection tasks of continuous quantity, such as length and duration. Lambrechts et al. (2013) concluded that continuous quantity processing skills were resilient to aging, likely due to their primitive basis (i.e., their appearance very early in development). One recent study directly investigated the impact of aging on non-symbolic numerical processing and ANS acuity. In order to study whether aging was associated with either refined or deteriorated basic numerical skills, Cappelletti et al. (2014) compared older and younger participants’ performance on a non-symbolic quantity discrimination task, similar to Halberda et al. (2012). Their results showed a larger mean *w* for older adults, initially suggesting a decline in ANS acuity with aging. However, further analyses suggested that this age difference was only present in trials requiring participants to inhibit perceptual information incongruent with numerosity (e.g., fewer but larger dots). As a consequence, the authors concluded that the observed decline in ANS acuity in aging may be accounted for by impaired inhibitory skills (Hasher and Zacks, 1988) rather than a decline in numerical skills.

As a result of sparse literature and variable methods, a clear conclusion cannot be drawn on the impact of aging on numerosity processing. The use of long presentation times in some studies is problematic, as they may encourage counting strategies. Therefore, these studies have likely measured counting rather than approximate, foundational numerical processing (e.g., Lemaire and Lecacheur, 2007; Gandini et al., 2008). Others fail to control for continuous perceptual variables such as size and cumulative area of stimuli (e.g., Gandini et al., 2008). Most research has also focused on a single measure to assess ANS performance (e.g., percentage correct in Lemaire and Lecacheur, 2007; *w* in Cappelletti et al., 2014). Finally, in Halberda et al.’s (2012) study suggesting a decline of ANS acuity in aging, trials requiring inhibitory control were not directly addressed, which may explain why the findings contradict those of Cappelletti et al. (2014). In the current study, we address these issues to gain a clearer understanding of the impact of aging on foundational non-symbolic numerical skills by: (a) using a short presentation time to clearly measure numerosity discrimination skills, rather than counting; (b) controlling for continuous perceptual variables with the introduction of congruent and incongruent trials, allowing further dissociation between impoverished numerosity skills and reduced inhibitory control (Cappelletti et al., 2014); and (c) using three measures (accuracy, RTs and *w*) to assess the global impact of aging on ANS acuity (Halberda et al., 2008). Moreover, since foundational numerical skills are not limited to non-symbolic processing, but are extended to acquired basic symbolic numerical skills, we also investigate whether aging and its life-long exposure to numbers is associated with refined or impaired foundational symbolic numerical skills.

Although the impact of aging on numerical cognition has recently begun to be investigated, it remains unclear whether such skills decline in aging. This question is still very much open in terms of basic symbolic and non-symbolic numerical skills. In all age populations, clear measures of ANS acuity have been used infrequently, which has potentially contributed to inconsistent results found in the literature (Gilmore et al., 2011; Price et al., 2012; Szűcs et al., 2013). In addition, direct investigation of basic symbolic abilities in aging has been largely ignored. The current study aims to measure the number sense, both non-symbolic (ANS) and symbolic (ENS) basic skills in younger and older adults, whilst controlling for mathematical achievement, general cognitive ability and years of education to establish whether basic numerical skills decline in aging (Duverne and Lemaire, 2004; Gandini et al., 2008; Lemaire and Arnaud, 2008; Li et al., 2010; Halberda et al., 2012), or remain stable (Lemaire and Lecacheur, 2007; Dormal et al., 2012; Lambrechts et al., 2013; Cappelletti et al., 2014). Firstly, we investigate non-symbolic numerical skills and ANS acuity using the non-symbolic numerical discrimination task Panamath, which has been standardized across thousands of participants (Halberda et al., 2012), a similar paradigm to that used recently in the literature on ANS acuity in aging (Cappelletti et al., 2014). Second, basic symbolic numerical skills are investigated with a symbolic comparison task, using simultaneously presented pairs of two-digit Arabic numbers (Nuerk et al., 2001). Both tasks will be investigated using defined age groups (older adults aged between 60 and 77, and younger adults between 19 and 25), with the findings adding to emerging research on the effect of aging on numerical cognition, particularly on basic symbolic and non-symbolic skills.

## Materials and Methods

### Participants

Fifty-two participants were recruited; 26 older adults between 60 and 77 (14 males; mean age = 65, SD = 4.5) and 26 younger adults between 19 and 25 (six males, mean age = 20.5, SD = 1.7). Participants were initially screened with an email questionnaire for history of psychiatric and neurological disorders, depression, and abnormal vision. Two participants’ data (one from each group) were removed due to a computer error. As a result, a total of 50 participants’ data were analyzed (25 per group). Participants in the older group were recruited voluntarily from the community, and participants in the younger group through the University of Hull Psychology Department. Amongst the younger group, 20 undergraduate students received course credit for participation. No payment was offered. Years of education were similar between groups, *p* > 0.6 (older adults: *M* = 16.5 years, SD = 2.6; younger adults: *M* = 16.2 years, SD = 1.7). The study was approved by the University of Hull Psychology Department ethics committee.

### Materials and Procedure

First, control measures were used. The Mini Mental State Exam (MMSE: Folstein et al., 1975; as in Gandini et al., 2009; Dormal et al., 2012) was administered to the older group, and the Geriatric Depression Scale (GDS: Yesavage et al., 1982) to all participants to rule out cognitive impairment and depression (depressed older adults are more likely to display cognitive changes than younger adults: Fiske et al., 2009). All older participants presented healthy MMSE scores over 27. Three participants from the older group presented a borderline score of 5 on GDS, and one participant from the younger group a score of 8, which could indicate depression. Participants presenting such scores were advised at debrief to seek help from their GP if they felt depressed. Their data were not excluded, as in comparing their performance (i.e., symbolic and non-symbolic RTs, accuracy and non-symbolic *w*) to the remainder of the group using a modified *t*-test (Crawford and Garthwaite, 2002), their results did not significantly differ (*p_s_* > 0.1). The spelling subtest of the Wide Range Achievement Test 4 part 2 (WRAT4: Wilkinson and Robertson, 2006) was used to control for general cognitive abilities (e.g., Castronovo and Göbel, 2012; Sasanguie et al., 2013). Forty two words increasing in difficulty were read out and presented within a sentence, with participants writing their answers. As in Castronovo and Göbel (2012), a calculation task based on the Graded Difficulty Arithmetic Test (Jackson and Warrington, 1986), was used to measure participants’ mathematic achievement index (MAI; overall percentage correct). This timed paper and pen task consisted of three sections, comprised of easy and difficult subsections: additions (a 30 s sub-section and a 90 s sub-section); subtractions (a 30 s sub-section and a 90 s sub-section); and multiplications with a 40 s sub-section and a 4 min sub-section. Participants answered as many questions as possible. Questions were presented and answered in written Arabic format.

Second, to investigate basic non-symbolic and symbolic numerical skills, we used two computerized tasks. Panamath was used to measure non-symbolic numerical abilities and ANS acuity (Halberda et al., 2008). In this well-known non-symbolic comparison task, participants must compare sets of yellow and blue dots presented simultaneously and judge which colored set is more numerous. To measure basic symbolic numerical abilities, a classic symbolic comparison task was used. In this task, participants decide which of two two-digit Arabic numbers is the largest (in magnitude; Nuerk et al., 2001). Both computerized tasks were conducted on an AMD Athlon computer (1280 × 1024 res), with participants sitting 50 cm away from the screen and responding using the keyboard.

Each participant took part in a single testing session split into two counterbalanced phases. In the first phase, participants undertook the MMSE, GDS and WRAT 4 spelling task, as well as the computerized non-symbolic comparison task. In the second phase, participants undertook the calculation task and the symbolic comparison task. Participants took short breaks between tasks, with testing complete within 2 h. Participants provided written informed consent before participation.

### Non-Symbolic Comparison Task

The stimuli were a set of blue and a set of yellow dots simultaneously presented for 200 ms (as in Halberda et al., 2012). The study utilized an online version of Panamath downloaded from www.panamath.org. Fast stimulus presentation was used (as in Halberda et al., 2008; Cappelletti et al., 2014) to reduce the likelihood of counting and the influence of working memory, increasing reliability in terms of directly testing the ANS (Maylor et al., 2008). Participants decided whether the blue or yellow dots were more numerous by pressing either ‘A’ or ‘L’ on the keyboard, which were covered with blue and yellow circles respectively. The yellow dots always appeared on the left of the screen, and the blue dots on the right. Total cumulative area of each dot array was adapted to control for the influence of perceptual variables: half the trials were non size-controlled, with the size of the average blue dot equal to that of the average yellow dot. Therefore, the more numerous set also had a larger total area (congruent trials). The other half of the trials were size-controlled, so that the number of blue pixels was equal to the number of yellow pixels regardless of numerosity (incongruent trials; Halberda et al., 2008). The size-controlled incongruent trials require inhibition skills, as participants must inhibit a response based on perceptual variables to attend to numerosity. Participants were asked to avoid counting and answer using their best impression. Each trial started with the participant pressing the space bar. Two sets of colored dots were then presented simultaneously for 200 ms. They were immediately followed by a color-matched backward mask (200 ms). A prompt then remained on-screen until an answer was provided. In half of the trials, the blue dots were more numerous, and in the other half the yellow dots were more numerous. Accuracy, RTs and the Weber fraction (*w*) for each participant were measured. Two trials were first used as practice trials. In total there were 384 trials, split into four ratio bins, as in Halberda and Feigenson’s (2008) study: Bin 1 = ratio 1.2; Bin 2 = ratio 1.3; Bin 3 = ratio 1.8; and Bin 4 = ratio 3. Ratio bins denote the ratio between the number of blue and yellow dots (ratio = bigger set/smaller set). For example, stimuli were categorized as being in Bin 1 (ratio 1.2) when 11 yellow dots and 13 blue dots were presented (13/11 = 1.2). According to Weber’s law, as the ratio increases, task difficulty decreases (Halberda et al., 2008). Dot numerosities ranged from 5 to 21 for each color. In the analysis, the effect of ratio and age group on ANS acuity (accuracy, RTs, and *w*) will be investigated.

### Symbolic Comparison Task

The symbolic comparison task was similar to that used by Nuerk et al. (2001). Stimuli comprised of a pair of two-digit numbers (e.g., 46 and 58), presented simultaneously (horizontally), in black font on a white background. Participants decided whether the larger number was on the left or right of the screen. The use of two-digit stimuli involves refined symbolic abilities, avoiding likely ceiling effects found in adults and older children when using single-digit stimuli (Lonnemann et al., 2011). The use of a larger numerical range should therefore allow greater discrimination between participants’ skills than in previous studies using a smaller numerical range (e.g., Cappelletti et al., 2014). Global distances between stimuli pairs were grouped into four distance bins indicating the total numerical distance between them: Bin 1 = distances [6–15]; Bin 2 = distances [16–24]; Bin 3 = distances [25–49]; Bin 4 = distances [51–71]. Task difficulty decreases with increasing distance bin. For example, the pair [31–81] has a larger global distance (distance of 50, Bin 4) than [31–51] (distance of 20, Bin 2), and therefore belongs to a larger distance bin. Stimuli ranged from 21 to 98. Participants responded as quickly as possible without sacrificing accuracy using the keyboard (A = left, L = right). The number on the left was larger in half the trials, and the number on the right was larger for the other half. There were 16 practice trials with feedback, followed by 240 randomized trials without feedback in two blocks. A black fixation cross appeared on a white background for 1000 ms, followed by stimuli until response (up to 3000 ms). Accuracy and RTs were recorded.

## Results

Group differences for control measures (GDS, Calculation task and Spelling task) were first investigated using independent *t*-tests. Older and younger adults’ mean *w_s_* were then compared using an independent *t*-test. ANOVAs were conducted on RTs and accuracy to assess the impact of aging on non-symbolic and symbolic numerical skills, with ratio/distance bin as a within-subjects factor and age group as a between-subjects factor. Additionally, in the non-symbolic comparison task, ANOVAs were conducted for each age group to determine the effect of trial type (congruent or incongruent) on RTs and accuracy. Where sphericity was violated, Greenhouse–Geisser corrections apply. Ratio effects for each participant were further analyzed by calculating individual regression slopes. Correlation analyses were conducted to determine whether mathematical achievement was related to basic symbolic and non-symbolic numerical skills. Finally, linear regression analyses further analyze the effect of age, education, and control measures on symbolic and non-symbolic skills.

### Control Measures

In the calculation task, positive correlations (*p_s_* < 0.001) were found between scores on the three sections (addition, subtraction, multiplication). For all participants, a MAI was determined by calculating the total percentage correct of all three sections combined. Independent-samples *t*-tests indicated that older adults (*M* = 90.55%, SD = 6.90) presented a significantly higher MAI than younger adults (*M* = 68.23%, SD = 15.38), *t*(33.3) = -6.62, *p* < 0.001. In the spelling task, older adults (*M* = 86.19%, SD = 8.59) also scored significantly higher than younger adults (*M* = 78.67%, SD = 5.97), *t*(48) = -3.60, *p* < 0.01. There were also no significant differences between groups on the GDS (*p* > 0.2). Overall, these results indicate that although both groups present similar levels of education, older people clearly demonstrate greater mathematics achievement. The results also reflect those of previous findings indicating greater mathematical abilities in older adults (Cappelletti et al., 2014), with greater spelling ability in aging also consistent with an observed increase in verbal knowledge across the lifespan (see Cappelletti et al., 2014 for vocabulary scores; Hedden and Gabrieli, 2004; Watson et al., 2005).

### Non-Symbolic Comparison Task

In the non-symbolic comparison task, ANS acuity was measured by accuracy, RTs, and *w* (Halberda et al., 2008). ANOVAs were conducted to assess the impact of age and ratio on RTs and accuracy. Further analyses were carried out to investigate the effect of aging on performance on congruent trials, where numerosity and perceptual continuous variables correlated (i.e., non size-controlled trials), and incongruent trials (i.e., size-controlled trials), where task irrelevant but salient information (e.g., cumulative area) had to be inhibited (Cappelletti et al., 2014).

First, an independent-samples *t*-test indicated that there was no significant difference in *w* between age groups, calculated after outlier removal (as below): *t*(48) = 0.146, *p* = 0.88. In both groups, the average *w* was 0.18, (older group SD = 0.182; younger group SD = 0.184), reflecting a similar *w* to that found in previous literature (Piazza et al., 2004).

**FIGURE 1:**
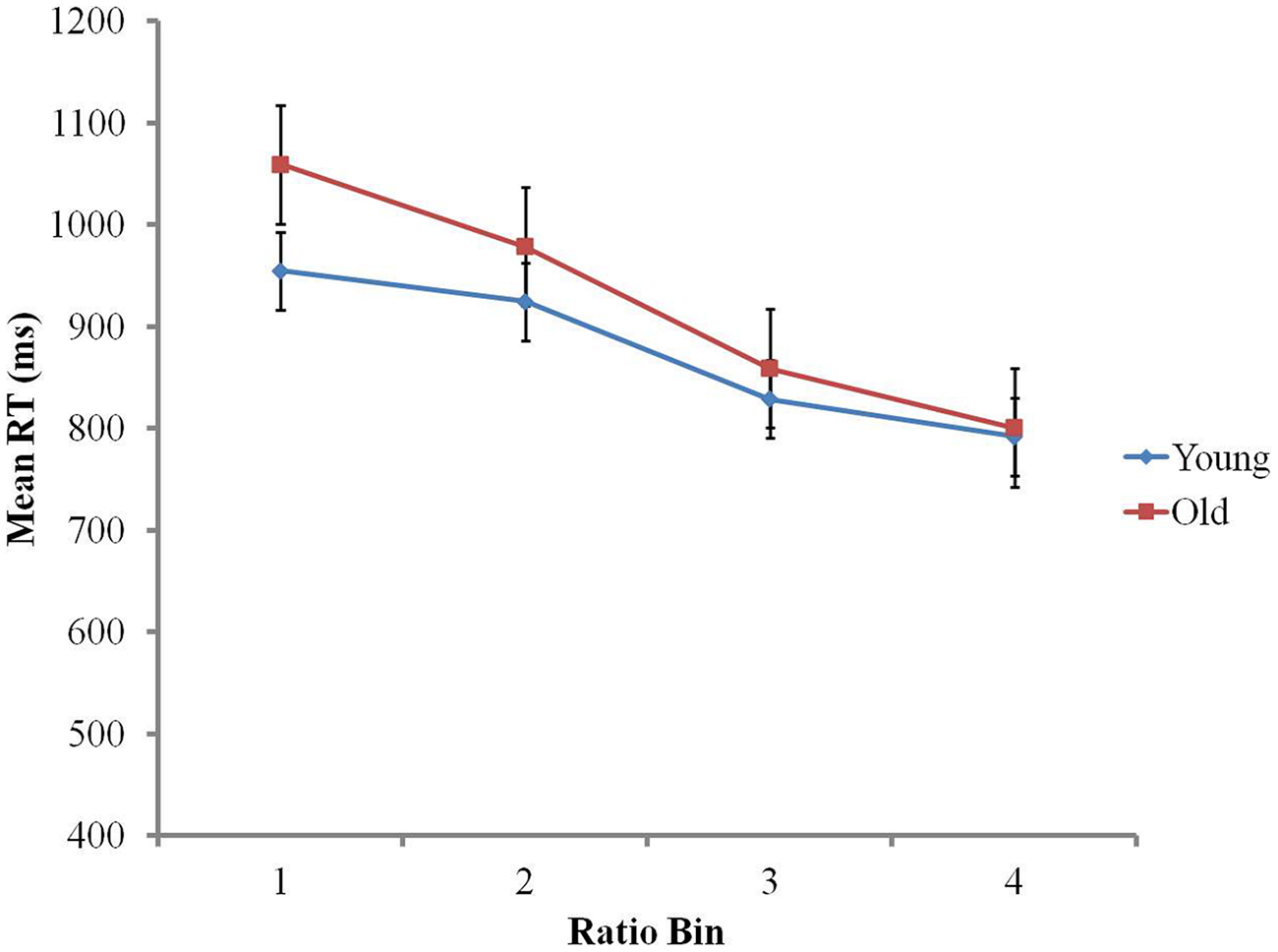
**The ratio effect on reaction times in the non-symbolic numerical comparison task**.

For further analyses, data were trimmed by applying a 3 SD cut-off for RTs on correct responses (2.11% of data removed). Preliminary analyses indicated that neither group showed a speed-accuracy trade-off (older adults *r* = 0.18, *p* > 0.38; younger adults *r* = 0.34, *p* = 0.1). To determine the effect of age and ratio on RTs, a 4 (ratio bin) × 2 (age group) mixed ANOVA was conducted, with ratio bin as a within-subject factor, and age group as a between-subject factor. Ratio bin had a main effect on RTs, *F*(1.52, 72.97) = 156.62, *p* < 0.001. This reflects the classic ratio effect: the larger the ratio, the faster the responses (Piazza and Izard, 2009). There was no main effect of age group on RTs, as both groups presented similar RTs, *F*(1,48) = 0.784, *p* > 0.3 (younger group *M* = 874 ms, SD = 308, older group *M* = 923 ms, SD = 360). However, the ratio bin × age group interaction was significant, *F*(1.52,72.97) = 7.21, *p* < 0.01 (see **Figure 1**). To further investigate this interaction, we computed each participant’s linear regression slope for RTs, with ratio bin as a predictor (De Smedt et al., 2009; Castronovo and Göbel, 2012). An independent *t*-test showed that the non-symbolic ratio effect was significantly more pronounced in the older group (Mean regression slope = -90.15, SD = 35.84) than the younger group (Mean regression slope = -58.70, SD = 38.85), *t*(48) = 2.98, *p* < 0.01.

**FIGURE 2:**
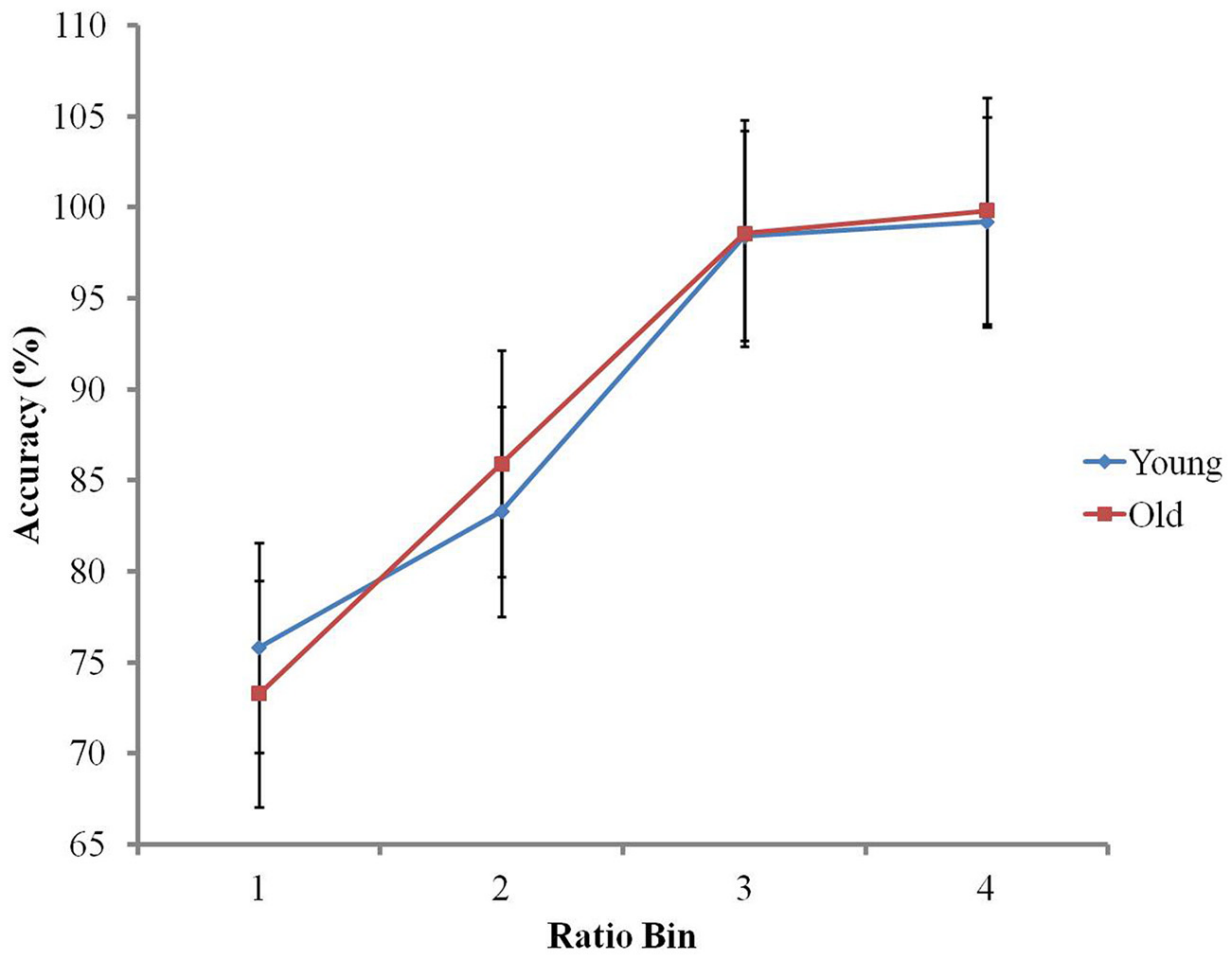
**The ratio effect on accuracy in the non-symbolic numerical comparison task**.

A 4 (ratio bin) × 2 (age group) ANOVA investigated ratio and age effects on non-symbolic accuracy. There was a main effect of ratio bin, *F*(1.96,93.94) = 474.2, *p* < 0.001, reflecting improving accuracy with increasing ratio (Bin 1 *M* = 74.59%, SD = 7.04, Bin 2 *M* = 84.62%, SD = 5.61, Bin 3 *M* = 98.50%, SD = 2.00, Bin 4 *M* = 99.52%, SD = 1.31; Piazza and Izard, 2009). There was no main effect of age group (*p* > 0.7), with both groups presenting similar accuracy (older group *M* = 89.57%, SD = 3.31, younger group *M* = 89.33%, SD = 2.97). The ratio bin × age group interaction was significant, *F*(1.96,93.94) = 3.57, *p* < 0.05. To further investigate this interaction, regression slopes were calculated for each participant, with ratio bin as a predictor of accuracy. An independent *t*-test showed that there was no significant difference between the groups’ regression slopes (*p* > 0.2). This indicates that the interaction cannot be accounted for by a global difference between the groups’ ratio effect on accuracy. Independent *t*-tests also showed that accuracy scores were similar in both groups in each ratio bin (*p_s_* > 0.1). Therefore, this interaction seems to be likely due to opposite patterns between the groups’ processing of ratio bins 1 and 2, with the younger group presenting slightly greater accuracy than the older group in ratio Bin 1 (*M* = 75.80%, SD = 6.06 in younger group; *M* = 73.38%, SD = 7.83 in older group), whilst the reverse can be found in Bin 2 (*M* = 83.31%, SD = 4.78 in younger group; *M* = 85.93%, SD = 6.16 in the older group; see **Figure 2**).

Correlation analyses were conducted between MAI and non-symbolic acuity (*w,* RTs and accuracy). Correlations between *w* and MAI (*p* > 0.2) and *w* and non-symbolic RTs (*p* > 0.1) did not reach significance. As expected, *w* correlated significantly with non-symbolic accuracy; *r* = -0.99, *p* < 0.001. Hierarchical regression analyses were conducted on RTs, accuracy, and *w* to investigate whether an effect of aging on non-symbolic ANS abilities might appear when controlling for possibly confounding variables (education, spelling performance, and MAI). Four steps were sequentially included in the analyses: (1) Education (years); (2) Spelling Score; (3) MAI; (4) Age Group; (5) Gender. All factors were non-significant predictors of RTs, accuracy and *w* (*p_s_* > 0.1).

These first results appear to show that non-symbolic foundational numerical skills are preserved in healthy aging. However, since recent data has suggested impaired numerosity discrimination in older participants when inhibition skills are required (i.e., in incongruent trials: Cappelletti et al., 2014), we conducted further analyses. In our study, we used both congruent trials where numerosity and total occupied area correlated (i.e., the larger the numerical set, the larger its cumulative area) and incongruent trials where each colored set occupied the same total area. To investigate possible decline in non-symbolic numerical skills in aging when inhibitory processes are required (Cappelletti et al., 2014), we ran a mixed ANOVA, with condition (congruent vs. incongruent) as a within-subjects variable and age group as a between-subjects variable on non-symbolic RTs, accuracy and *w*. In line with Cappelletti et al. (2014), we found a main effect of condition on *w,* with incongruent trials resulting in reduced ANS acuity (higher mean *w* = 0.20, SD = 0.06), compared to congruent trials (*w* = 0.16, SD = 0.04), *F*(1,48) = 39.34, *p* < 0.001. However, our results indicated that this condition effect on *w* was similar for both age groups (*p* > 0.7), with no interaction (*p* > 0.6). On RTs, there were no main effects of condition (congruent trials mean RT = 897 ms, SD = 199, incongruent = 901 ms, SD = 200; *p* > 0.2) or age group (*p* > 0.3; mean RT older group = 923 ms, SD = 360, younger group *M* = 874 ms, SD = 309). Nevertheless, the condition × group interaction on RTs was marginally significant (*p* = 0.065), since older adults presented slower RTs in incongruent trials (mean RT = 929 ms, SD = 202) compared to congruent trials (mean RT = 919 ms, SD = 200), *t*(24) = -2.22, *p* < 0.05. On the other hand, younger participants presented similar RTs in both conditions (*p* > 0.6). Participants were more accurate during congruent trials (mean accuracy = 91%, SD = 3.12) than during incongruent trials (mean accuracy = 87%, SD = 3.91), *F*(1,48) = 47.28, *p* < 0.001. There was, however, no main effect of age group (*p* > 0.7), and no interaction (*p* > 0.2). Our results indicate that participants’ non-symbolic performance declined in terms of *w* and accuracy regardless of age group in incongruent trials where inhibition of task-irrelevant perceptual information was necessary compared to during congruent trials, where numerosity and area correlated. However, in line with Cappelletti et al.’s (2014) results, healthy aging tends to be associated with a somewhat more pronounced decline in numerosity discrimination skills where inhibition is required, since the older group were significantly slower during incongruent trials compared to congruent trials, with younger adults showing no such effect.

### Symbolic Comparison Task

**FIGURE 3:**
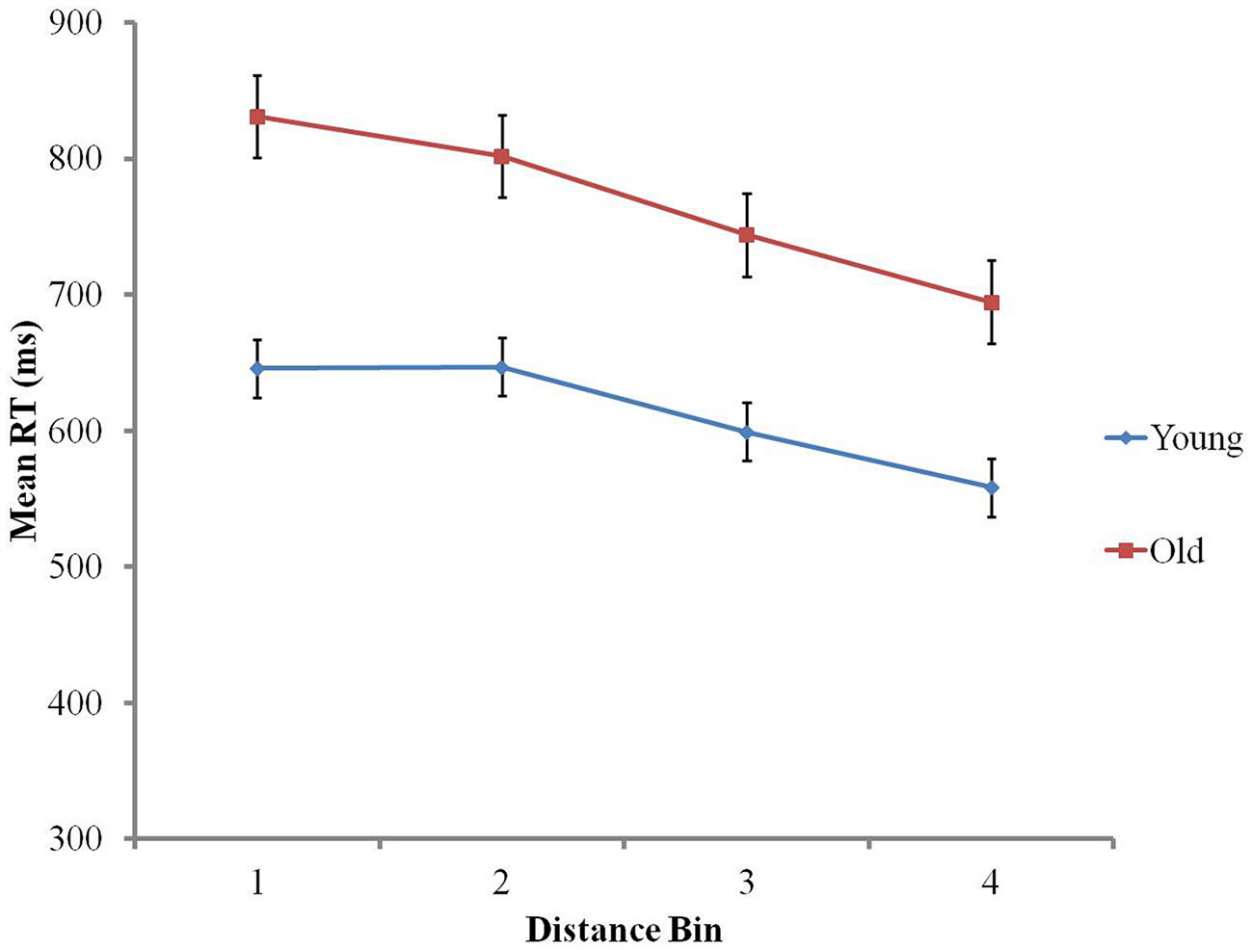
**The distance effect on reaction times in the symbolic numerical comparison task**.

In order to investigate the impact of aging on basic symbolic numerical skills, analyses were conducted on RTs and accuracy. RT data were trimmed by applying the same 3 SD cut-off as in the non-symbolic comparison task (1.45% data removed). Neither group presented a speed-accuracy trade off (older adults: *r* = 0.25, *p* > 0.2; younger adults: *r* = 0.36, *p* = 0.1). On RTs, distance and age group effects were investigated using a 4 (distance bin) × 2 (age group) mixed ANOVA. Distance had a main effect on RTs: the larger the distance, the faster the response, as explained by Weber’s law, *F*(2.09,100.13) = 290.23, *p* < 0.001 (Piazza and Izard, 2009). Younger adults were significantly faster (mean RT = 613 ms, SD = 72) than older adults (mean RT = 768 ms, SD = 94), *F*(1,48) = 44.97, *p* < 0.001. The distance bin × age group interaction was also significant, *F*(2.09,100.13) = 12.94, *p* < 0.001. This interaction appears to be mainly due to the fact that younger participants had similar RTs for distance bins 1 and 2 (mean RT = 645 ms, SD = 79; mean RT = 648 ms, SD = 78 respectively; *p* > 0.6). On the contrary, older participants’ RTs differed across all distance bins, including bins 1 and 2, illustrated by significantly slower RTs in distance Bin 1 (mean RT = 831 ms, SD = 104), than distance Bin 2 (mean RT = 802 ms, SD = 95), *F*(1,24) = 22.34, *p* < 0.001 (see **Figure 3**).

**FIGURE 4:**
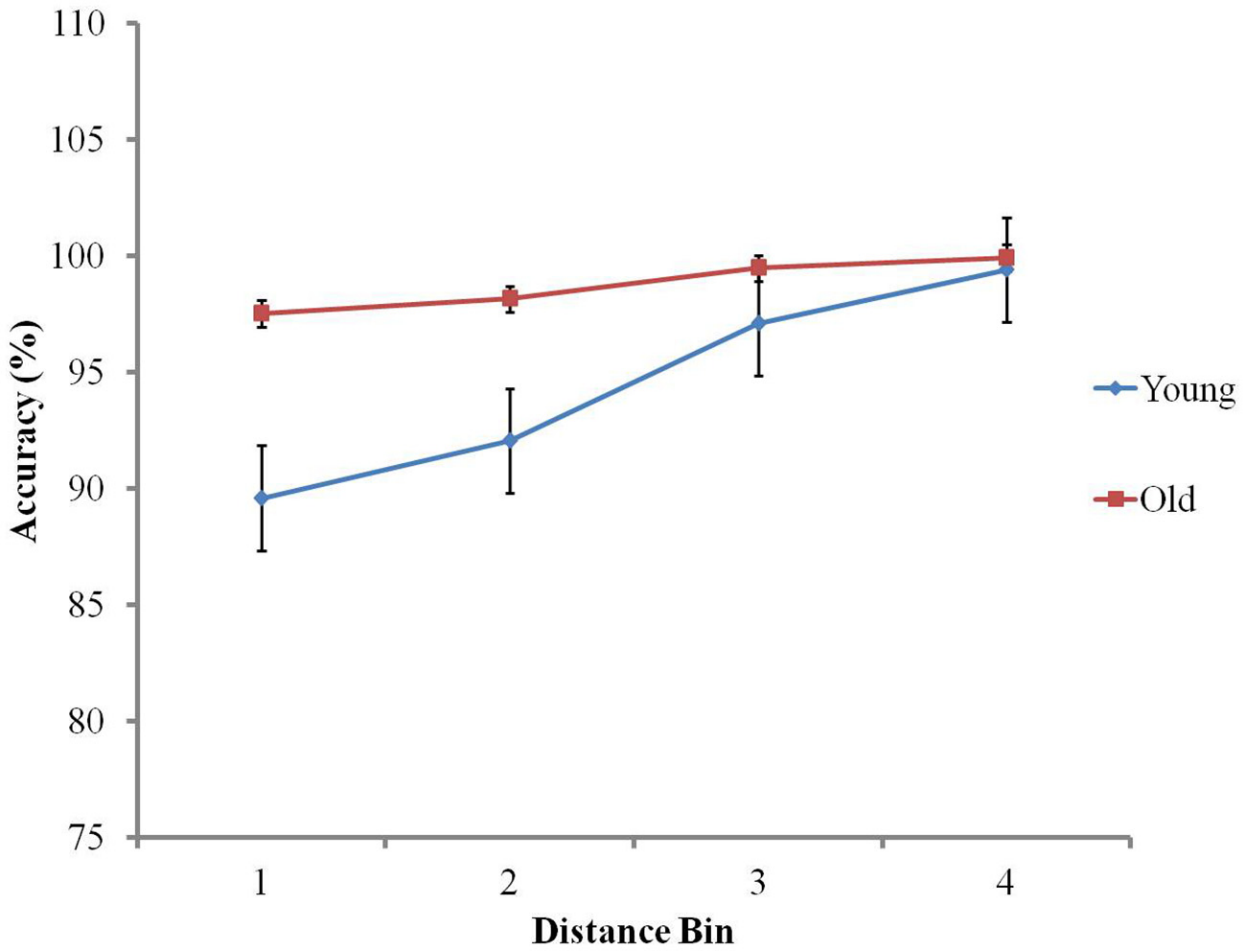
**The distance effect on accuracy in the symbolic numerical comparison task**.

Similar analyses of accuracy show a main effect of distance in accordance with Weber’s law, *F*(2.13,102.25) = 53.46, *p* < 0.001: as global distance increases, so does accuracy (mean accuracy Bin 1 = 93.57, SD = 6.01, Bin 2 = 95.10, SD = 4.24, Bin 3 = 98.29, SD = 2.48, Bin 4 = 99.66, SD = 0.78; Piazza and Izard, 2009). There was also a main effect of age group, as older adults (*M* = 99%; SD = 2) were significantly more accurate than younger adults (*M* = 95%; SD = 3), *F*(1,48) = 62.48, *p* < 0.001. The interaction between distance and age group was significant, *F*(2.13,102.25) = 19.36, *p* < 0.001 (see **Figure 4**). Individual regression slope analysis showed that the symbolic distance effect on accuracy was significantly more pronounced in the younger group (mean regression slope = 3.44) than the older group (mean regression slope = 0.85), *t*(48) = 6.46, *p* < 0.001.

As in the non-symbolic comparison task, correlation analyses were conducted between MAIs and symbolic RTs and accuracy. Correlations between MAI and symbolic RTs (*r* = 0.32, *p* < 0.05) and accuracy (*r* = 0.63, *p* < 0.001) were significant. Further, hierarchical regression analyses were conducted on RTs and accuracy to determine the effect of age group on symbolic processing when controlling for education, spelling, MAI and gender. Education, spelling scores and gender were non-significant predictors of symbolic performance (*p_s_* > 0.3). Both MAI and age group were significant predictors of RTs (β = -0.38, *p* < 0.05, β = 0.90, *p* < 0.001 respectively). Age strongly predicted RTs, accounting for 42% of the variance, Δ*F*(1,45) = 41.73, *p* < 0.001. Crucially, being in the older group was the only significant predictor of higher accuracy (β = 0.60, *p* < 0.001). On all measures, age group accounted for a significant additional percentage of variance, Δ*F*(1,45) = 20.91, *p* < 0.001), particularly for accuracy (19%). Overall, age affected symbolic comparison abilities over and above any other factor, most notably MAI.

## Discussion

The impact of healthy aging on basic numerical abilities has only recently begun to be researched. In the field of numerical cognition, much attention has been paid to the number sense in children, and its link with mathematical achievement (Halberda et al., 2008; De Smedt et al., 2009; Libertus et al., 2012, 2013; Piazza et al., 2013). Where investigation has turned to aging, studies have mostly focused on higher-level numerical skills (e.g., Duverne and Lemaire, 2004; Lemaire and Arnaud, 2008). Those which have researched non-symbolic abilities find contradictory results, present methodological limitations (e.g., variable presentation times of stimuli, little or no control of perceptual variables), and tend to focus on a single measure of ANS acuity, such as *w.* Some studies have found a decline in non-symbolic numerical abilities with age (Halberda et al., 2012), whereas others have found no such effect once age-related inhibitory decline was controlled for (Cappelletti et al., 2014). In terms of basic symbolic abilities, findings are sparse and methods limited, with a focus on high-level arithmetical skills or the use of small numerical ranges in comparison tasks. Overall, some studies suggest a decline in symbolic numerical processing in aging (e.g., Duverne and Lemaire, 2004; Lemaire and Arnaud, 2008), whereas others suggest preserved (but potentially slower) symbolic abilities in older adults (Cappelletti et al., 2014). More importantly, aging and basic symbolic numerical skills appear not to have been directly investigated in the literature. As a result, no clear conclusion can be drawn on the effect of aging on basic numerical processes, both non-symbolic and symbolic. The current study addresses these issues in being the first to study both non-symbolic and symbolic numerical skills as basic foundational skills in aging. Our findings suggest that aging does not have a detrimental effect on basic non-symbolic numerical processing, whilst it seems to have a positive effect on basic symbolic numerical processing and mathematical achievement. Additionally, our results further support previous research showing a positive effect of aging on verbal knowledge (Hedden and Gabrieli, 2004; Cappelletti et al., 2014), since aging appeared to be associated with superior spelling performance.

The current findings on aging and foundational non-symbolic numerical skills support recent research suggesting that healthy aging is associated with the preservation of non-symbolic quantity processing (both continuous; Dormal et al., 2012; Lambrechts et al., 2013; and discrete; Lemaire and Lecacheur, 2007; Cappelletti et al., 2014), as non-symbolic discrimination skills were preserved in the older group. These results are in line with the suggestion that basic numerical skills may be resilient to cognitive aging as they stem from a primitive, innate system originating from evolutionary abilities (Feigenson et al., 2004; Lambrechts et al., 2013). Previous contradictory results suggesting a decline of ANS acuity in aging are likely to result from different methodological issues. For example, Halberda et al. (2012) concluded that ANS acuity decreases steadily from age 30. However, the study’s older group shows a large variability in age range (45–85 years), which could have led to a large variability in performance. Additionally, as highlighted by Cappelletti et al. (2014), no distinction was made by Halberda et al. (2012) between congruent and incongruent trials. Moreover, as the validity of ANS tasks using congruent and incongruent trials has recently been questioned (Szűcs et al., 2013), further research is required to investigate to what extent the current and previous findings may be affected by task design.

Our results, in line with previous findings (Li et al., 2010; Cappelletti et al., 2014), support the idea that inhibition skills should be considered when assessing numerosity discrimination skills, particularly in older groups. Indeed, our data on trials involving inhibition skills (incongruent trials) indicate that all participants were affected when continuous perceptual variables were incongruent with numerosity, as both groups were less accurate and presented larger mean *w*_s_. These findings reflect previous research demonstrating the impact of continuous perceptual variables on numerosity judgments (Hurewitz et al., 2006; Dakin et al., 2011; Gebuis and Reynvoet, 2012a). Moreover, supporting the findings of Cappelletti et al. (2014), aging appears to be associated with greater sensitivity to interference from incongruent continuous variables, as older participants were somewhat slower in incongruent trials than congruent trials, whilst younger participants presented similar RTs regardless of congruency. This pattern of response times in the older group may represent impaired inhibitory control of a response based on area in order to respond to numerosity (e.g., Cappelletti et al., 2014). However, that both younger and older participants in our study, rather than older participants alone as in Cappelletti et al. (2014), presented declined accuracy and higher *w* in incongruent trials may be accounted for by the use of a backward mask in the current study to eliminate short-term memory representations of stimuli. The absence of a backward mask in Cappelletti et al.’s (2014) study may have benefited younger participants, as they have stronger short-term memory abilities than older adults (Salthouse and Babcock, 1991), and therefore a superior ‘after image’ (Sperling, 1960) of stimuli, resulting in less interference from incongruent trials. Another methodological difference was that Cappelletti et al. (2014) used intermixed displays of dots, whereas separate, simultaneous arrays were used in the current study (as in Fuhs and McNeil, 2013; Gilmore et al., 2013; Smets et al., 2013). Recent research has suggested that differences in stimulus display methods may lead to unreliable ANS measures (Hurewitz et al., 2006; but see Price et al., 2012; Szűcs et al., 2013). Intermixed displays during a short presentation time could be more difficult for older participants due to their reduced useful field of view (Lemaire and Lecacheur, 2007), likely leading to poorer performance on the more difficult incongruent trials. Our results on incongruent trials are therefore somewhat in line with Cappelletti et al. (2014), supporting the hypothesis that previous findings of impaired numerosity discrimination in aging may reflect inhibitory decline, rather than impoverished non-symbolic numerical processing *per se*. Overall, our findings on non-symbolic numerical comparison further support the conclusion that aging does not affect basic non-symbolic numerical abilities, possibly as a result of the innate nature of the ANS (Feigenson et al., 2004; Lambrechts et al., 2013).

Secondly, our study highlights a positive impact of aging on basic symbolic numerical skills, as well as mathematical achievement. With the introduction of a larger numerical range, we were able to study symbolic numerical skills in aging, whilst avoiding likely ceiling effects of using single-digit stimuli (Lonnemann et al., 2011). Our data indicate that aging appears to be associated greater symbolic numerical abilities. Although older participants presented slower RTs due to processing-speed decline in aging (Salthouse, 1996), they were more accurate than younger participants, with a less pronounced symbolic distance effect on accuracy. These results extend Cappelletti et al.’s (2014) primary observations of preserved basic symbolic numerical abiltities in aging on a smaller numerical range. Altogether, these findings suggest the presence of a better anchored and more precise symbolic numerical representation, as well as greater mathematical achievement in aging, as a result of lifetime experience with numbers. The results are also in line with the suggestion that symbolic discrimination abilities improve with age (Salthouse and Kersten, 1993; De Smedt et al., 2013; Cappelletti et al., 2014).

Symbolic and non-symbolic distance effects have been found to be affected by individual number knowledge, with mathematical achievement being negatively associated with the symbolic distance effect in children (e.g., De Smedt et al., 2009; Holloway and Ansari, 2009) and adults (e.g., Castronovo and Göbel, 2012), but positively correlated with the non-symbolic distance effect (in children Gilmore et al., 2010; in adults, Castronovo and Göbel, 2012). Likewise, in the current study, participants in the older group presented greater mathematical achievement, as well as a decreased symbolic distance effect on accuracy and an increased non-symbolic distance effect on RTs compared to the younger group. The results therefore give further support to the assumption that longer lifetime exposure to numbers is associated with greater mathematical knowledge, as well as a better defined symbolic number system and a greater tendency to automatically transcode non-symbolic numerosities into their corresponding symbolic representations (Halberda et al., 2008; Gilmore et al., 2010; Castronovo and Göbel, 2012).

Further to our findings regarding the effect of aging on basic non-symbolic and symbolic numerical abilities, the link between non-symbolic abilities and mathematical achievement was investigated, as it represents a key theme in numerical cognition research, particularly in early development. Non-symbolic acuity and MAI did not correlate in either age group, reflecting recent findings (Inglis et al., 2011; Castronovo and Göbel, 2012; Price et al., 2012; but see Libertus et al., 2012), and reinforcing the hypothesis that ANS acuity may reach a maximum in adulthood, reducing the strength of a link between the ANS and mathematical ability in adults (Castronovo and Göbel, 2012). Alternatively, mathematical achievement may only correlate with symbolic abilities, both in children (Holloway and Ansari, 2008, 2009) and adults (Castronovo and Göbel, 2012), a prediction further supported by the current findings, as well as neuropsychological evidence of a relationship between specific brain regions used for both basic and advanced symbolic numerical processing (Ansari et al., 2006; Cantlon et al., 2006; Piazza et al., 2007; Izard et al., 2008). Therefore, by directly investigating foundational non-symbolic and symbolic numerical skills in aging, our study provides further evidence that the ANS corresponds to a primitive number system resilient to aging, as well as being unrelated to education and increased practice with age (Feigenson et al., 2004; Castronovo and Göbel, 2012; Lambrechts et al., 2013). On the other hand, the ENS, similarly to other education-related abilities, such as spelling and vocabulary (Hedden and Gabrieli, 2004), benefits from life-long exposure and practice associated with aging. Improved basic symbolic processing and mathematical ability in the older group may possibly reflect a generational, qualitative difference in terms of mathematical education (Geary and Lin, 1998). However, regression analyses suggest that this is unlikely, as increasing age accounted for significantly more variance in symbolic discrimination accuracy than mathematical achievement.

The current study further addresses the question of the impact of aging on cognition in general (Hedden and Gabrieli, 2004), suggesting that foundational numerical processing may be one of a few cognitive skills along with verbal memory, implicit memory, and emotional processes to be preserved in healthy aging. Our results bring to light the effect of cognitive aging on basic symbolic and non-symbolic numerical abilities. However, difficulties with using non-symbolic comparison tasks which control only limited perceptual variables to measure ANS acuity (Gebuis and Reynvoet, 2012b; Szűcs et al., 2013) mean that further research is required utilizing newer non-symbolic numerosity comparison paradigms controlling other perceptual variables (e.g., Gebuis and Reynvoet, 2011). The current findings could inform research into the effects of pathological aging on numerical cognition, such as in AD (Girelli et al., 1999; Kaufmann et al., 2002; Duverne et al., 2003; Maylor et al., 2005; Delazer et al., 2006; Khodarahimi and Rasti, 2011). As numerical cognition is largely dependent on the parietal lobes (Piazza and Izard, 2009; Roitman et al., 2012), and these regions of the brain undergo significant atrophy early in the course of AD (Jacobs et al., 2011, 2012; Bruner and Jacobs, 2013) compared to normal aging (Dixon et al., 2004), basic numerical tasks could prove a useful diagnostic tool. Such application may be advantageous as the tasks used in the current study are straightforward to apply. Moreover, as the measures remain stable in healthy aging, their use may provide a specific marker for AD. The discovery of differences in basic numerical skills between healthy older adults and those with AD could assist in the detection of the disease at the earliest stages, which is vital in developing more effective treatments and creating better outcomes (Salthouse, 2009). In particular, the non-symbolic comparison task used in this study (Panamath: Halberda et al., 2008) has been used with populations of varying abilities, including very young children, demonstrating its flexibility of application and therefore the potential for use in clinical populations.

## Author Contributions

JN, JC, WM, and CG substantially contributed to the conception and design of the study; JN collected and analyzed the data; JN and JC substantially contributed to the interpretation of the data; JN and JC substantially contributed to drafting the work; JN, JC, WM, and CG substantially contributed to revising the work critically for important intellectual content; JN, JC, WM, and CG gave final approval for the current version of the paper to be submitted for publication; JN, JC, WM, and CG agree to be accountable for all aspects of the work.

## Conflict of Interest Statement

The authors declare that the research was conducted in the absence of any commercial or financial relationships that could be construed as a potential conflict of interest.
